# Mid-Term Evolution of the Serum Acylcarnitine Profile in Critically Ill Survivors: A Metabolic Insight into Survivorship

**DOI:** 10.3390/nu15163595

**Published:** 2023-08-16

**Authors:** Anne-Françoise Rousseau, Arsène Ngongan, Camille Colson, Pauline Minguet, Sarah Neis-Gilson, Etienne Cavalier, Grégory Minguet, Benoit Misset, François Boemer

**Affiliations:** 1 Intensive Care Department and Burn Centre, University Hospital of Liège, University of Liège, 4000 Liège, Belgium; 2 GIGA-Research, GIGA-I3 Thematic Unit, Inflammation and Enhanced Rehabilitation Laboratory (Intensive Care), University of Liège, 4000 Liège, Belgium; 3 Clinical Chemistry Department, University Hospital, University of Liège, 4000 Liège, Belgium; 4 Anesthesiology Department, University Hospital, University of Liège, 4000 Liège, Belgium; 5 Biochemical Genetics Lab, Department of Human Genetics, University Hospital, University of Liège, 4000 Liège, Belgium

**Keywords:** carnitine, critical illness, survivors, mitochondrial dysfunction, catabolism, muscle

## Abstract

It is unknown if the abnormal acylcarnitine (AC) profile observed early after discharge of a prolonged stay in an intensive care unit (ICU) would persist over time. This prospective observational study aimed to describe the mid-term AC profile evolution in survivors of a prolonged ICU stay (≥7 days). Adults enrolled in our post-ICU follow-up program and who attended the consultation 3 months (M3) after discharge were included. Serum AC concentrations were assessed within 7 days following ICU discharge (T0) and at M3. A total of 64 survivors were analyzed after an ICU stay of 15 (9–24) days. Free carnitine (C0) concentration decreased from 45.89 (35.80–127.5) to 28.73 (20.31–38.93) µmol/L (*p* < 0.001). C0 deficiency was not observed at T0 but in 7/64 (11%) survivors at M3. The total AC/C0 ratio (normal ≤ 0.4) was 0.33 (0.24–0.39) at T0 and reached 0.39 (0.30–0.56) at M3 (*p* = 0.001). A ratio >0.4 was observed in 16/64 (25%) at T0 and in 32/64 (50%) at M3 (*p* = 0.006). The short-chain ACs decreased from 1.310 (0.927–1.829) at T0 to 0.945 (0.709–1.127) µmol/L at M3 (*p* < 0.001). In parallel, the urea/creatinine ratio and the Sarcopenic Index, respectively, decreased and increased between T0 and M3. This AC profile is suspected to signal a mitochondrial dysfunction and was, especially for short-chain ACs, a marker of protein catabolism.

## 1. Introduction

Physical dysfunction is a frequent disabling complaint among patients who survived a stay in an intensive care unit (ICU) [1,2]. Patients may report a reduced physical capacity up to five after the ICU stay [3,4]. When there is no plausible etiology other than the critical illness itself, its treatments and the ICU hospitalization, this functional impairment is called “ICU-acquired weakness”. This syndrome is a complex association of muscle wasting, the impaired homeostasis of myofibrillar proteins, changes in muscle composition, impaired regeneration, derangements in excitation–contraction coupling and acquired mitochondrial dysfunction [5,6].

The main role of carnitine is the transport of long-chain fatty acids from the cytoplasm into the mitochondria for the beta-oxidation of fatty acids [7]. Carnitine also binds acyl residues deriving from the intermediary metabolism of amino acids to facilitate their elimination. These functions result in carnitine esterification into acylcarnitine derivatives. The endogenous carnitine pool is comprised of L-carnitine (the physiologically active isomer of carnitine) and acylcarnitines (AC). The L-carnitine is the major representative of this pool: the normal acylcarnitine to L-carnitine (AC/C0) ratio is around 0.25 [8]. A ratio exceeding 0.4 in the blood is thought to represent a disturbed mitochondrial metabolism [8,9].

Carnitine deficiency has been associated with muscle weakness. Critically ill patients may be considered at risk of secondary deficiency [10] in the case of prolonged undernutrition, prolonged parenteral nutrition (commercial formulations do not contain carnitine), prolonged continuous renal replacement therapy (carnitine is lost in the effluent) or valproate treatment (carnitine is used for the urinary excretion of drug derivates) [9,11].

In addition to carnitine deficiency, critically ill patients may have a significant impairment of mitochondrial function [12]. The role of mitochondrial dysfunction in chronic muscle weakness after sepsis has been highlighted in animal studies [13]. A similar mitochondrial dysfunction in skeletal muscles has been suggested in recent studies assessing exercise tolerance in ICU survivors using cardiopulmonary exercise testing [14,15]. Mitochondrial dysfunction leads to a deficit in fatty acid β-oxidation, with an accumulation of non-oxidized fatty acyl-CoA esters into the mitochondria. These esters will further be conjugated to carnitine (mainly as long-chain AC).

In a previous cohort of survivors of a prolonged ICU stay, the AC profile was altered during the week after ICU discharge, compared to a reference adult population [16]. An excess in short-chain AC was observed, and more than a quarter of the population had an increased AC/C0 ratio. However, the evolution of the AC profile during the post-ICU trajectory is still unknown. The aim of the present observational study was to describe the AC profile of prolonged-ICU-stay survivors during the mid-term post-ICU period.

## 2. Materials and Methods

### 2.1. Patients

Our six-unit adult intensive care academic department includes 44 beds. Patients who survived an ICU stay of ≥7 days are routinely invited to our post-intensive care follow up. Patients with an end-of-life condition, coma or known dementia, and patients unable to communicate in French (local language), are not invited. If we are unable to give them information about the follow up before hospital discharge, they are considered as lost to follow up. The first contact occurs during the first 7 days following ICU discharge, with a nurse-led face-to-face standardized visit assessing acute functional disorders. Face-to-face consultations are then scheduled at 1, 3 and 12 months after ICU discharge. These consultations are cancelled in the case of ongoing hospitalization in acute care or in inpatient rehabilitation facilities. These consultations are standardized, addressing physical status and functional performances, nutritional status and body composition, bone health, mental health disorders, cognitive impairment, sleep disorders and health-related quality of life. At each timepoint, a blood analysis focused on inflammation and metabolic biomarkers (including the acylcarnitine profile) is part of our standard analysis.

All consecutive patients discharged from an ICU after a stay of ≥7 days between November 2020 and February 2022, included in the follow-up trajectory according to the above-described criteria, and who attended the consultation 3 months after discharge were enrolled in the present study. Further exclusion criteria were known primary carnitine deficiency or L-carnitine supplementation. Clinical data and biological parameters were prospectively recorded after the visit in the ward (T0) and the consultation (M3).

In accordance with Belgian law and the opinion of the Ethics Committee of the University Hospital of Liege (Chair: Pr Vincent Seutin; local reference: 2020/424), informed consent was not required because the study did not modify patients’ management and the data were anonymously collected. 

### 2.2. Serum Acylcarnitine Profiling

The biological data were generated from one single laboratory (Unilab, CHU de Liège) accredited according to ISO 15.189 guidelines.

**Figure 1:**
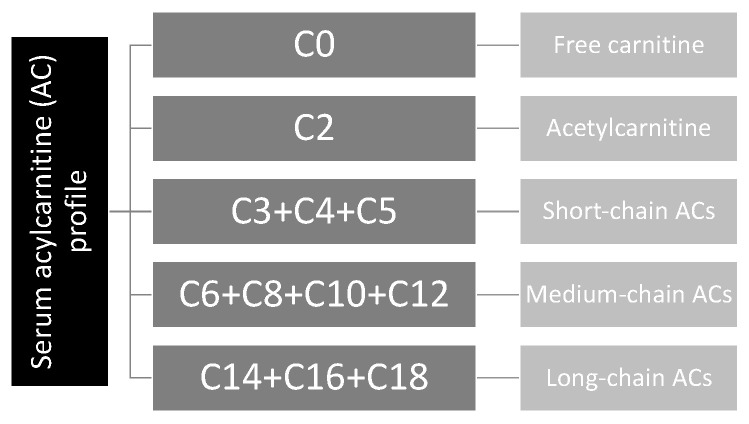
Acylcarnitine profile.

In the text, Cx refers to the number of carbons in the acyl chain of carnitine derivatives (for instance, if x = 2, it refers to 2 carbons in the acyl chain). The ACs are classified as follows: (1) acetylcarnitine (C2) and short-chain ACs (SCACs: C3 + C4 + C5), (2) medium-chain ACs (MCACs: C6 + C8 + C10 + C12), (3) long-chain ACs (LCACs: C14 + C16 + C18), (4) hydroxylated ACs (OH-ACs) and (5) dicarboxylic ACs (DC-ACs) (Figure 1).

Blood samples were collected through a central or peripheral venous line placed for clinical use, or through venous punction. Blood was drawn into a serum gel tube (BD Vacutainer, Becton, Dickinson and Company, Franklin Lakes, NJ, USA), before being centrifuged (3500 rpm, 15 min and 4 °C). A supernatant was frozen at −20 °C and stored for a later analysis.

Serum AC concentrations (free carnitine (C0), C2-, C3-, C3DC-, C4-, C5-, C5:1-, C5DC-, C6, C6-DC-, C8-, C8:1-, C10-, C10:1-, C10:2-, C12-, C12:1-, C14-, C14:1-, C14:2-, C14-OH-, C16-, C16:1-, C16-OH-, C16-OH-, C18-, C18:1-, C18:2-, C18-OH-, C18:1-OH- and C18:2-OH-carnitine) were determined using a method described in a previous publication [17]. 

Reference ranges, established in the same lab, referred to a population of 50 apparently healthy (and non-hospitalized) individuals aged from 18 to 81 years (55 (43–62.5) years), including 46% males (23/50). In this population, there was no difference in the AC profile between males and females. 

### 2.3. Other Data

Demographic data and data related to the ICU stay were retrospectively extracted from medical charts.

The following blood markers were also collected prospectively at the two timepoints: blood white cell count (Sysmex XN Series, Norderstedt, Germany), C-reactive protein (CRP), albumin, prealbumin, triglycerides, urea, enzymatic creatinine (Alinity C, Abbott, Lake Bluff, IL, USA) and cystatin C (Cobas, Roche, Mannheim, Germany). The glomerular filtration rate (eGFR) was estimated using the creatinine-based CKD-EPI equation. The ratio of serum urea/creatinine was calculated: an elevated ratio reflects muscle catabolism [18]. The Sarcopenia Index was defined as [(serum creatinine/serum cystatin C) × 100], with a low index reflecting a reduced muscle mass [19]. 

### 2.4. Analysis

A statistical analysis was performed using Graphpad Prism (version 9.0 for Mac OSX, Graphpad Inc., San Diego, CA, USA). Normality was assessed using the Shapiro–Wilk test. Data were expressed as medians with lower and upper quartiles (Q1–Q3) for quantitative parameters. Qualitative variables were represented using a count and percent. Comparisons between data were made using the Wilcoxon signed-rank test and Fisher’s exact test, as required. A *p* value < 0.05 was considered statistically significant. 

## 3. Results

**Figure 2:**
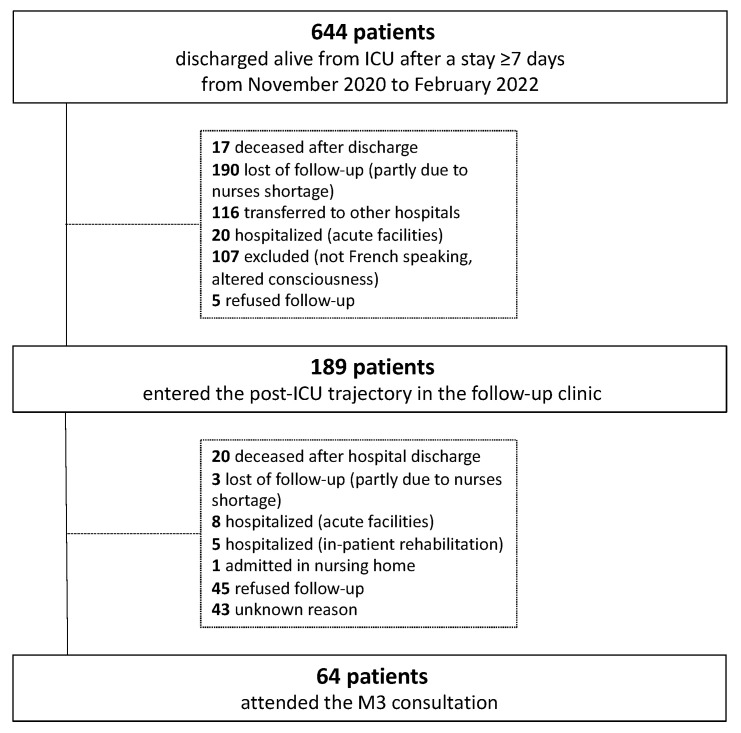
Flow chart.

Between November 2020 and February 2022, 644 patients were discharged alive from the ICU after a stay of ≥7 days. Of these patients, 189 were assessed at T0. Finally, 64 attended the M3 consultations and were analyzed (Figure 2). 

The characteristics of the included subjects are detailed in Table 1. Age, weight and BMI did not differ between males and females (*p* = 0.986, *p* = 0.333 and *p* = 0.169, respectively). Similarly, the proportions of primary failures were similar in males and females. Only one patient was treated for a human immunodeficiency virus infection. No patients received valproate during the ICU stay or after discharge. The maximum CRP and creatinine blood concentrations observed during the ICU stay were, respectively, 224.8 (171.2–321.7) mg/L and 1.35 (0.89–2.26) mg/dL. 

AC profiles at both timepoints (T0 and M3) are detailed in Table 2. There were significant changes in the AC profile over time. C0 (free carnitine) concentration significantly decreased between T0 and M3: no patients had C0 deficiency at T0, while 7/64 patients (10.9%) had a C0 concentration < centile 2.5 of the reference population at M3 (*p* = 0.013). The sum of short-chain AC significantly decreased at M3 compared to T0: respectively, 0.94 (0.71–1.13) μmol/L and 1.31 (0.93–1.83) μmol/L (*p* < 0.001). The long-chain ACs were higher than in the reference population (0.59 (0.38–0.83) μmol/L), but stable over time: 0.81 (0.58–1.07) μmol/L and 0.82 (0.58–1.02) μmol/L, respectively, in T0 and M3 (*p* = 0.580). The AC/C0 ratio increased between T0 (0.33 (0.24–0.39)) and M3 (0.39 (0.30–0.56)) (*p* = 0.001) and was higher than in the reference population (0.25 (0.17–0.35)) at both timepoints. A ratio >0.4 was observed in 16/64 (25%) patients at T0. This proportion reached 50% (32/64) at M3 (*p* = 0.006).

The other blood biomarkers measured at the same timepoints are represented in Table 3. Creatinine at T0 did not statistically differ between males and females (respectively, 0.79 (0.63–1.37) and 0.66 (0.43–0.99) mg/dL, *p* = 0.074). The blood concentration of CRP and the white cell count significantly decreased at M3 compared to T0. This was associated with a significant increase in albumin and prealbumin concentrations. The triglyceride concentrations were stable over time. An increase in creatinine concentration was observed at M3 compared to T0, in parallel with a decrease in eGFR. On the contrary, cystatin C concentration was stable over time. The urea/creatinine ratio decreased while the Sarcopenic Index increased between T0 and M3.

## 4. Discussion

In the present cohort of ICU survivors, significant changes in the AC profile were observed during the mid-term period following ICU discharge.

As reported in our previous cohorts [16,17], carnitine (C0) deficiency was not observed during the week after ICU discharge. However, the prevalence of C0 deficiency reached more than 10% of the patients at M3. The underlying reason is unclear. Drug therapies could be reasonably ruled out, as deficient patients were not treated with medications inducing C0 deficiency. Survivors remain at risk of malnutrition in the post-ICU period, from both quantitative and qualitative points of view [20], and malnutrition is a known risk factor for carnitine deficiency [21]. However, the impact of potential undernutrition on C0 status has not been studied in ICU survivors.

Half of the patients presented an elevated AC/C0 ratio at M3, while they were only a quarter at T0. This was related to a progressive decrease in C0, when LCACs remained stable although higher than in the reference population. These observations probably reflect mitochondrial dysfunction. Indeed, it is commonly accepted that increased LCAC levels are associated with impaired fatty acid oxidation in mitochondria [3], and an AC/C0 ratio exceeding 0.4 in the blood is attributed to a disturbed mitochondrial metabolism [8,9]. A similar metabolic signature has already been observed in cohorts assessed during the first hours after ICU admission for sepsis and correlated with a higher 28-day mortality [22,23]. During the recovery phase after severe COVID-19 pneumonia, exercise intolerance was suspected to be related to persistent inflammation and oxidative stress, and subsequent lower fatty acid oxidation in mitochondria [15]. In that study, ICU survivors underwent cardiopulmonary exercise testing. They suffered from a slowed and incomplete recovery, without signs of cardiac or ventilatory failure, but associated with a hypermetabolic status at rest and a disproportionate oxygen consumption during exercise. Persistent inflammation, based on increased myeloperoxidase blood concentrations, was suspected to explain these altered exercise patterns with metabolic disorders. In an attempt to improve these metabolic disorders, carnitine has been supplemented using the L-carnitine isomer. In a small, randomized controlled trial including critically ill patients, a carnitine supplementation (3 g daily for 7 days) during the acute phase of illness reduced inflammation markers [24]. At lower doses during a prolonged period, L-carnitine supplementation could also decrease inflammation markers in diseases characterized by chronic inflammation [25]. In older adults, a supplementation with L-carnitine (with a daily dose of 4.5 g) during 6 months, in association with a moderate-intensity exercise program, resulted in an increase in the carnitine content in muscles and the total fat oxidation during muscle training [26]. From a toxicological point of view, a daily chronic dose of 2 g of L-carnitine is considered safe. L-carnitine supplementation could be promising for ICU survivors, but to the best of our knowledge, has never been tested in this clinical setting.

In this cohort, SCACs were elevated at T0 compared with the reference population, but significantly decreased at M3. SCACs are in part the result of the degradation of branched-chain amino acids (BCAA). In particular, BCAA are important energy substrates in muscle during periods of stress [27]. The acyl-CoA derivates, generated with the catabolism of BCAA, will transfer their acyl group to carnitine to form C3 and C5 ACs [28]. Increased levels of SCACs could thus be considered as a marker of protein catabolism. Accordingly, in the present study, the decrease in SCACs was associated with a decrease in the urea/creatinine ratio (reflecting protein catabolism) and an increase in the Sarcopenic Index (reflecting muscle mass). In a recently published observational study on ICU survivors, we showed that SCAC concentrations in ICU survivors increased with the ICU length of stay and illness severity, with increased concentrations in patients surviving a prolonged ICU stay compared to a shorter stay [23]. All these observations suggest that SCACs could serve as catabolic markers, especially when urea/creatinine or the Sarcopenic Index are not suitable because of kidney failure. However, this hypothesis needs further confirmation. A practical application of SCAC measurement would be the monitoring of the catabolic/anabolic balance of amino acids following critical illness, with the hope of improving therapeutic interventions with nutritional, pharmacological or rehabilitation strategies [29]. However, to date, the value of SCACs as such a biomarker requires further investigations.

This study has some limitations. First, the number of patients in our cohort was limited. Nonetheless, our post-ICU clinic ensured a standardized follow up, limiting the risk of missed data. Furthermore, the AC profile was assessed using mass spectrometry, providing the concentrations of C0 and the entire range of its acyl-esters. This approach is expected to have strengthened the validity of the present results. Second, the evolution of the catabolic/anabolic balance can be assessed by measuring muscle strength (with a dynamometer) and muscle mass (using bio-impedance). Mitochondrial dysfunction can be approached with clinical tools such as cardiopulmonary exercise testing or inferred biologically with biomarkers of oxidative stress. Although these methods could have been used to confirm the present findings about muscle catabolism and mitochondrial dysfunction, they were not available or feasible on a systematic basis at the time of our patient follow up. Finally, it would have been informative to know the pre-ICU (baseline) status of our patients, but such evaluation is, by definition, challenging as ICU admissions are usually unexpected.

## 5. Conclusions

Up to 3 months after a prolonged ICU stay, significant changes in the AC profile were observed compared to the immediate post-discharge period. The observations related to AC/C0, SCACs and LCACs suggest prolonged metabolic derangements combining mitochondrial dysfunction and branched-chain amino acid catabolism. The role of AC as a source and/or marker of muscle dysfunction commonly described in ICU survivors should be further specified.

## Figures and Tables

**Table 1:** Demographics of the cohort.

Data		*n* = 64
Male, *n* (%)		44 (68.7)
Age, y		63 (50.2–68.7) Males: 63 (50.2–68.8) Females: 62 (50.5–69.2)
Weight, kg		77 (69.1–90) Males: 79.1 (69.6–95) Females: 74.2 (69–85.5)
BMI, kg/m^2^		26.9 (23.8–30.6) Males: 26 (23.1–30.3) Females: 28.6 (25.3–33.2)
Active smoking, *n* (%)		10 (15.6)
Admission category, *n* (%)	Medical	30 (46.9)
	Surgical	34 (53.1)
Primary dysfunction, *n* (%)	Cardiovascular	24 (37.5)
	Pulmonary	15 (23.4)
	Neurologic	6 (9.4)
	Digestive and hepatic	8 (12.5)
	Polytrauma	1 (1.6)
	Other	10 (15.6)
Simplified Acute Physiology Score II		33.5 (26–53.7)
Mechanical ventilation (MV) >24 h, *n* (%)		48 (75)
MV duration, d		7 (2–16.8)
Renal replacement therapy (RRT), *n* (%)		4 (6.2)
RRT duration, d		10.5 (5.7–28)
Extracorporeal membrane oxygenation, *n* (%)		3 (4.7)
Propofol-based sedation, *n* (%)		46 (71.9)
Propofol duration, d		6 (2–12)
Type of nutrition during ICU stay, *n* (%)	Oral nutrition	34 (53.1)
	Enteral nutrition	40 (62.5)
	Parenteral nutrition	10 (15.6)
ICU LOS, d		15 (9.2–23.8)
Hospital LOS, d		43 (26–67.5)

Data are expressed as medians with lower and upper quartiles (Q1–Q3). BMI: body mass index; ICU: intensive care unit; LOS: length of stay; MV: mechanical ventilation; RRT: renal replacement therapy.

**Table 2:** Acylcarnitine concentration in the two timepoints.

Acylcarnitines (μmol/L)	Reference Range	T0	M3	*p* Value
C0	14.95–84.34	45.89 (35.8–127.5)	28.73 (20.31–38.93)	<0.001
C2	2.71–21.28	10.81 (7.48–14.85)	8.61 (6.65–12.90)	0.019
Short-chain ACs				
C3	0.086–3.329	0.540 (0.431–0.809)	0.337 (0.220–0.477)	<0.001
C3-DC	0.007–0.221	0.065 (0.037–0.106)	0.060 (0.038–0.080)	0.1465
C4	0.038–0.400	0.235 (0.170–0.385)	0.160 (0.112–0.240)	<0.001
C5	0.024–0.202	0.100 (0.066–0.150)	0.080 (0.059–0.110)	0.002
C5:1	0.004–0.043	0.020 (0.008–0.030)	0.020 (0.010–0.030)	0.913
C5-OH	0.011–0.073	0.050 (0.040–0.080)	0.050 (0.032–0.060)	0.011
C5-DC	0.021–0.267	0.150 (0.096–0.233)	0.146 (0.110–0.200)	0.719
Medium-chain ACs				
C6	0.011–0.164	0.098 (0.066–0.148)	0.053 (0.040–0.087)	<0.001
C6-DC	0.019–0.578	0.058 (0.030–0.114)	0.045 (0.030–0.080)	0.006
C8	0.016–0.291	0.100 (0.070–0.138)	0.086 (0.050–0.120)	0.021
C8:1	0.019–0.331	0.164 (0.114–0.287)	0.152 (0.110–0.265)	0.635
C10	0.023–0.622	0.110 (0.081–0.187)	0.100 (0.072–0.160)	0.269
C10:1	0.016–0.265	0.074 (0.085–0.099)	0.062 (0.050–0.080)	0.091
C10:2	0.004–0.050	0.020 (0.010–0.029)	0.020 (0.010–0.021)	0.917
C12	0.011–0.239	0.050 (0.040–0.067)	0.058 (0.040–0.090)	0.139
C12:1	0.012–0.253	0.060 (0.040–0.094)	0.047 (0.030–0.070)	0.027
Long-chain ACs				
C14	0.008–0.081	0.035 (0.027–0.050)	0.040 (0.030–0.060)	0.208
C14:1	0.016–0.315	0.071 (0.050–0.101)	0.060 (0.040–0.080)	0.181
C14:2	0.005–0.080	0.022 (0.020–0.031)	0.020 (0.020–0.030)	0.747
C14-OH	0.002–0.016	0.010 (0.007–0.014)	0.010 (0.010–0.020)	0.641
C16	0.060–0.293	0.190 (0.140–0.267)	0.190 (0.141–0.267)	0.514
C16:1	0.007–0.154	0.040 (0.030–0.056)	0.035 (0.023–0.047)	0.093
C16-OH	0.001–0.009	0.010 (0.004–0.020)	0.010 (0.010–0.010)	0.336
C18	0.019–0.082	0.075 (0.040–0.110)	0.086 (0.055–0.120)	0.166
C18:1	0.048–0.479	0.210 (0.151–0.317)	0.210 (0.148–0.260)	0.169
C18:2	0.012–0.106	0.070 (0.046–0.110)	0.070 (0.050–0.090)	0.430
C18:1-OH	0.001–0.010	0.010 (0.004–0.020)	0.010 (0.005–0.020)	0.587
C18:2-OH	0.001–0.006	0.010 (0.002–0.010)	0.010 (0.003–0.010)	0.755

Data are expressed as medians with lower and upper quartiles (Q1–Q3). Cx refers to the number of carbons in the acyl chain of carnitine derivatives (see the Materials and Methods section).

**Table 3:** Ancillary biochemical parameters.

Biomarkers	Reference Range	T0	M3	*p* Value
White blood cells (/mm^3^)	10^3^/mm^3^	9.57 (7.14–12.84)	7.48 (6.25–9)	0.004
C-reactive protein (mg/L)	0–5	33.1 (16.3–85.5)	2.8 (1–10.9)	<0.001
Albumin (g/L)	32–46	32 (29–35)	43 (39–44)	<0.001
Prealbumin (g/L)	0.2–0.4	0.20 (0.15–0.27)	0.27 (0.24–0.31)	0.075
Triglycerides (mg/dL)	<175	143 (99–176)	141.5 (102.8–242.5)	0.277
Urea (mg/dL)	15–55	41.9 (29.1–55.7)	34.1 (26.2–56.5)	0.293
Creatinine (mg/dL)	0.55–1.02 (females) 0.55–1.18 (males)	0.78 (0.59–1.27)	0.95 (0.74–1.26)	0.023
eGFR (mL/min/1.73 m^2^)	>60	96.5 (55.5–114.3)	75 (53.5–100)	0.013
Urea/Creatinine		46.1 (35.6–69.5)	37.8 (27.4–50.7)	0.008
Cystatin C (mg/L)	0.62–1.11	1.28 (1.03–1.59)	1.38 (1.12–1.72)	0.648
Creatinine/Cystatin C		52 (45–76)	70 (59–85)	0.057

Data are expressed as medians with lower and upper quartiles (Q1–Q3). eGFR: estimated glomeral filtration rate.

## Data Availability

The datasets used and/or analyzed during the current study are available from the corresponding author on reasonable request.
